# Demonstration Of Age-Related Increase In Blood-Brain Barrier Permeability By Longitudinal Intravital Microscopy

**DOI:** 10.1093/geroni/igab046.2503

**Published:** 2021-12-17

**Authors:** Adam Nyul Toth, Stefano Tarantini, Jordan DelFavero, Feng Yan, Priya Balasubramanian, Qinggong Tang, Anna Csiszar, Zoltan Ungvari

**Affiliations:** 1 University of Oklahoma Health Sciences Center, Oklahoma City, Oklahoma, United States; 2 University of Oklahoma, Norman, Oklahoma, United States

## Abstract

Age-related blood-brain barrier disruption and cerebromicrovascular rarefaction contribute importantly to the pathogenesis of both vascular cognitive impairment and dementia (VCID) and Alzheimer's disease (AD). Recent advances in geroscience research enable development of novel interventions to reverse age-related alterations of the cerebral microcirculation for prevention of VCID and AD. To facilitate this research there is an urgent need for sensitive and easy-to-adapt imaging methods, which enable longitudinal assessment of changes in BBB permeability and brain capillarization in aged mice, that could be used in vivo to evaluate treatment efficiency. To enable longitudinal assessment of changes in BBB permeability in aged mice equipped with a chronic cranial window, we adapted and optimized two different intravital two-photon imaging approaches. By assessing relative fluorescence changes over the baseline within a volume of brain tissue, after qualitative image subtraction of the brain microvasculature, we confirmed that in 24 month old C57BL/6J mice cumulative permeability of the microvessels to fluorescent tracers of different molecular weights (0.3 kDa to 40 kDa) is significantly increased as compared to that of 5 month old mice. Real-time recording of vessel cross-sections showed that apparent solute permeability of single microvessels is significantly increased in aged mice vs. young mice. Cortical capillary density, assessed both by intravital two-photon microscopy and optical coherence tomography (OCT) was also decreased in aged mice vs. young mice. The presented methods have been optimized for longitudinal (over the period of 36 weeks) in vivo assessment of cerebromicrovascular health in preclinical geroscience research.

